# TPD54 Downregulates ERK Signaling in Oral Squamous Cell Carcinoma Cells

**DOI:** 10.3390/biomedicines14102295

**Published:** 2026-10-09

**Authors:** Konomi Yamada, Yoshiki Mukudai, Maki Nara, Masataka Watanabe, Nodoka Kindaichi, Toshikazu Shimane, Tatsuo Shirota, Seigo Ohba

**Affiliations:** Department of Oral and Maxillofacial Surgery, School of Dentistry, Showa University, 2-1-1 Kitasenzoku, Ota-ku 145-8515, Tokyo, Japan; gd23-k027@dent.showa-u.ac.jp (K.Y.); gd22-m017@dent.showa-u.ac.jp (M.N.); dt2091watanabe@dent.showa-u.ac.jp (M.W.); dt2155kindaichi@dent.showa-u.ac.jp (N.K.); shima-tskz@dent.showa-u.ac.jp (T.S.); tshirota@dent.showa-u.ac.jp (T.S.); sohba@dent.showa-u.ac.jp (S.O.)

**Keywords:** tumor protein D52 family, tumor protein D54, squamous cell carcinoma, oral cancer

## Abstract

**Background/Objectives:** Epidermal growth factor receptor is frequently overexpressed in oral squamous cell carcinoma (OSCC) and is associated with poor prognosis. We previously demonstrated that members of the tumor protein D (TPD) 52 family regulate the malignant behavior of OSCC cells and that epidermal growth factor (EGF) induces TPD52 expression. In this study, we investigated the relationship between the TPD52 family and EGF signaling, focusing on TPD54. **Methods:** SAS OSCC cells were treated with EGF and gefitinib, and the expression of TPD52 family members (TPD52, TPD53, and TPD54) was analyzed using reverse transcription-quantitative polymerase chain reaction (RT-qPCR) and Western blotting. Nascent RNA and RNA degradation assays were conducted to investigate the mechanisms regulating TPD52 family expression. TPD54-overexpressing and TPD54-knockdown cells were used to evaluate EGF signaling, cell cycle progression, proliferation, apoptosis, autophagy, migration, and invasion. **Results:** EGF stimulation increased the mRNA expression of all TPD52 family members. Nascent RNA analysis revealed increased transcription, whereas RNA degradation assays indicated reduced mRNA stability. TPD54 overexpression attenuated EGF-induced ERK phosphorylation and suppressed EGF-induced cell cycle progression. However, under the present experimental conditions, TPD54 had little effect on cell proliferation, apoptosis, autophagy, migration, or invasion. **Conclusions:** TPD54 is an EGF-responsive protein that suppresses EGF-induced ERK activation and attenuates cell cycle progression in OSCC cells. Although TPD54 exerted minimal effects on cell proliferation, apoptosis, autophagy, invasion, or migration under the tested conditions, these findings suggest that TPD54 functions as a negative regulator of EGF signaling and provide insight into its molecular role in OSCC.

## 1. Introduction

Oral squamous cell carcinoma (OSCC) is the most common malignancy of the maxillofacial region, accounting for more than 90% of all oral cancers, and is frequently associated with poor prognosis because of its highly invasive and metastatic nature [1]. Although surgery, radiotherapy, and chemotherapy are all established treatment options, the overall survival rate has remained largely unchanged over the past three decades, highlighting the need for new therapeutic targets [2].

The tumor protein D (TPD) 52 family includes TPD52, TPD53, TPD54, and TPD55 [3]. TPD52, the founding member, is overexpressed in several malignancies, including breast and lung cancers [3,4]. In addition, TPD52 family members, except for TPD55, exhibit aberrant expression in multiple cancers, including colorectal, ovarian, and prostate cancers [5,6,7]. In OSCC, we previously demonstrated that TPD52 is highly expressed in both OSCC tissues and adjacent hyperplastic epithelium [8]. We also showed that TPD54 negatively regulates extracellular matrix-dependent migration and adhesion of OSCC cells [9], and that the balance between TPD52 and TPD54 expression influences the proliferation, invasion, metastasis, and survival of highly malignant OSCC cells under hypoxic conditions [10].

Members of the TPD52 family—comprising TPD52, TPD53, TPD54, and TPD55—are small, conserved proteins characterized by coiled-coil motifs that mediate both homo- and heteromeric interactions [11,12]. Accumulating evidence has indicated that TPD52 family members function as key regulators of intracellular membrane trafficking and organelle integrity [13,14]. These proteins localize to various membrane compartments, including the Golgi apparatus, endosomes, and intracellular nanovesicles (INVs), where they interact with Rab GTPases and SNARE complexes to facilitate vesicle transport and membrane fusion [14,15]. Furthermore, TPD52 family proteins, particularly TPD52, play essential roles in regulating protein secretion, including exocytosis and endolysosomal secretion in secretory tissues [16,17]. Furthermore, these proteins have been directly implicated in lipid metabolism by regulating lipid accumulation and lipid droplet biogenesis, thus contributing to cellular energy homeostasis [18,19]. While these conserved trafficking and metabolic functions are shared across the family, emerging studies have indicated that individual TPD52 family members also exert distinct, isoform-specific roles in cancer progression (Supplementary Figure S1).

Epidermal growth factor receptor (EGFR) is frequently overexpressed in OSCC and is associated with poor prognosis [20]. As a tyrosine kinase receptor, EGFR regulates cell proliferation, differentiation, survival, and migration through multiple downstream signaling pathways, including the RAS/RAF/mitogen-activated protein kinase kinase (MEK)/extracellular signal-regulated kinase (ERK) and PI3K/AKT signaling cascades [21]. We previously demonstrated that epidermal growth factor (EGF) stimulation upregulates TPD52 expression in OSCC cells; however, whether EGF similarly regulates other TPD52 family members remains unclear [22].

Based on these findings, we hypothesized that TPD52 family members would participate in EGF-mediated proliferation and invasion of OSCC cells. Therefore, we investigated the relationship between the TPD52 family and EGF signaling in SAS cells, with a particular focus on the role of TPD54 in regulating downstream signaling and tumor cell behavior. The present study revealed that TPD54 is an EGF-responsive molecule that negatively regulates EGF-induced ERK signaling and cell cycle progression in OSCC cells.

## 2. Materials and Methods

### 2.1. Cell Culture

The human OSCC cell line SAS [8,10,23] (JCRB accession number, JCRB0260), derived from human tongue squamous cell carcinoma, was kindly provided by Dr. Ochiya (Tokyo Medical University, Tokyo, Japan). Cells were cultured in high-glucose Dulbecco’s modified Eagle’s medium (FUJIFILM Wako Pure Chemical Corporation, Osaka, Japan) supplemented with 10% fetal bovine serum, 100 U/mL of penicillin, and 100 μg/mL of streptomycin. The cells were maintained at 37 °C in a humidified incubator containing 5% CO_2_ (pHcbi, MCO-170AIC-PJ, PHC Corporation, Tokyo, Japan) [8,10,14]. For EGF stimulation, cells were treated with recombinant human EGF (1.0 ng/mL; Sigma-Aldrich, SRP3027, St. Louis, MO, USA). EGFR signaling was inhibited using gefitinib (0.5 μM; Sigma-Aldrich, SML1657, St. Louis, MO, USA). Unless otherwise indicated, experiments were performed under four conditions: control, EGF, gefitinib, and EGF plus gefitinib.

### 2.2. Antibodies

Rabbit polyclonal antibodies against TPD52 (#27648-1-AP), TPD53 (#14732-1-AP), and TPD54 (#11795-1-AP) were purchased from Proteintech (Rosemont, IL, USA). Rabbit monoclonal antibodies against phospho-MEK1/2 (p-MEK1/2, #2338), MEK1/2 (#9126), phospho-ERK1/2 (p-ERK1/2, #4370), ERK1/2 (#4695), p62 (#39749), GAPDH (#2118), and LC3 (#12741) were purchased from Cell Signaling Technology (Danvers, MA, USA). Rabbit polyclonal anti-HaloTag antibody (G9281) was purchased from Promega (Madison, WI, USA). Horseradish peroxidase-conjugated goat anti-rabbit IgG secondary antibody (ECL Anti-Rabbit IgG, Horseradish Peroxidase-linked Whole Antibody, NA934V) was purchased from Cytiva (Marlborough, MA, USA). The details of each antibody are shown in Table 1.

### 2.3. RNA Extraction and Reverse Transcription-Quantitative Polymerase Chain Reaction

**Figure 1:**
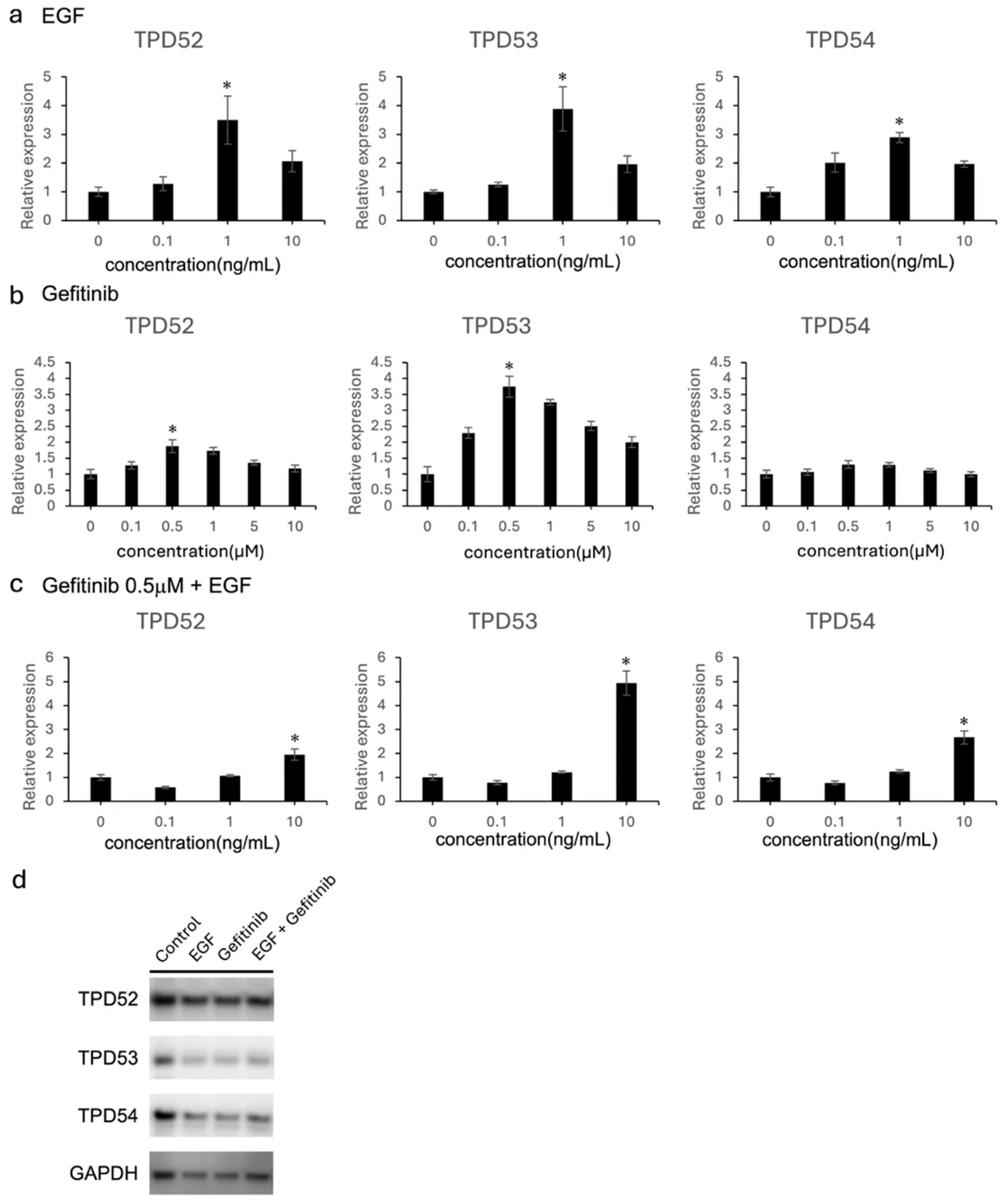
Effects of EGF and gefitinib on mRNA (a, b and c) and protein expression (d) of TPD52 family members. (**a**) Relative mRNA expression levels of *TPD52*, *TPD53*, and *TPD54* following stimulation with EGF (0, 0.1, 1, and 10 ng/mL) for 24 h. (**b**) Relative mRNA expression levels of *TPD52*, *TPD53*, and *TPD54* following stimulation with gefitinib (0, 0.1, 0.5, 1, 5, and 10 μM) for 24 h. (**c**) Relative mRNA expression levels of *TPD52*, *TPD53*, and *TPD54* following EGF stimulation (0, 0.1, 1, and 10 ng/mL) and 0.5 μM of gefitinib. (**d**) Representative Western blot images of TPD52, TPD53, TPD54, and GAPDH. RT-qPCR data are presented as relative expression levels normalized to the corresponding 0 ng/mL or 0 mM group (set to 1). Data are presented as the mean ± SD from three independent experiments (*n* = 3). * *p* < 0.05 versus 0 ng/mL or 0 mM group.

To determine the optimal treatment conditions, SAS cells were exposed to EGF (0, 0.1, 1, or 10 ng/mL), gefitinib (0, 0.1, 0.5, 1, 5, or 10 μM), or EGF (0, 0.1, 1, or 10 ng/mL), in combination with 0.5 μM of gefitinib for 24 h before RNA extraction. Based on these results, 1.0 ng/mL of EGF and 0.5 μM of gefitinib were selected for subsequent experiments (Figure 1).

Total RNA was isolated using TRIzol reagent (Life Technologies, Carlsbad, CA, USA) according to the manufacturer’s instructions and stored at −80 °C. Total RNA (100 ng) was reverse-transcribed into cDNA using a ReverTra Ace qPCR RT Kit (TOYOBO, Osaka, Japan) according to the manufacturer’s instructions [8]. Reverse transcription–quantitative polymerase chain reaction (RT-qPCR) was performed using THUNDERBIRD Probe qPCR Mix (TOYOBO) according to the manufacturer’s protocol under the following cycling conditions: 95 °C for 1 min, followed by 49 cycles of 95 °C for 15 s and 60 °C for 30 s. TaqMan Gene Expression Assays (Thermo Fisher Scientific, Waltham, MA, USA) were used for *TPD52* (Hs00893105), *TPD53* (Hs00914766), *TPD54* (Hs00900580), and *ACTB* (Hs01060665_g1). Amplification was performed using a CFX Connect Real-Time PCR Detection System (Bio-Rad, Hercules, CA, USA) [24]. Relative gene expression levels were calculated using the 2^−ΔΔCt^ method and normalized to *ACTB* expression [25].

### 2.4. Gene Transfection

Cells were seeded in 6-well tissue culture plates at a density of 1 × 10^6^ cells/well and transfected 24 h later. TPD54 knockdown was performed using small interfering RNA (siRNA) targeting human TPD54 (EHU077061; Sigma-Aldrich), while a firefly luciferase-targeting siRNA (EHUFLUC) (Sigma-Aldrich) served as the negative control. For overexpression experiments, cells were transfected with a HaloTag-TPD54 expression vector (pFN21AE2584) or the corresponding HaloTag control vector (G659) (Promega). Transfections were performed using Lipofectamine 2000 (Thermo Fisher Scientific) according to the manufacturer’s instructions and previous reports [8,23].

### 2.5. Protein Extraction and Western Blotting

For drug treatment experiments, SAS cells were cultured under each experimental condition for 24 h prior to protein extraction. For overexpression and knockdown experiments, transfected cells were reseeded into 24-well tissue culture plates 24 h after transfection, cultured overnight, and treated under each experimental condition for an additional 24 h before protein extraction, as previously described [10,23]. For Western blotting, 20 μg of total protein was separated by sodium dodecyl sulfate–polyacrylamide gel electrophoresis using 4–20% gradient gels (Bio-Rad) and transferred onto polyvinylidene difluoride membranes using the iBlot 2 Dry Blotting System (Thermo Fisher Scientific). Membranes were blocked with Tris-buffered saline (Takara Bio, Shiga, Japan) containing 0.2% skim milk (Cell Signaling Technology) for 1 h at room temperature and incubated sequentially with the appropriate primary and secondary antibodies as previously described [24]. Protein bands were detected using Amersham ECL Prime Western Blotting Detection Reagent (GE Healthcare UK Ltd., Buckinghamshire, UK) and visualized using a ChemiDoc XRS Plus Imaging System (Bio-Rad). Band intensities were quantified using ImageJ software (version 1.54d; NIH, Bethesda, MD, USA) and normalized to GAPDH. Membranes were stripped using stripping solution (FUJIFILM Wako Pure Chemical Corporation) according to the manufacturer’s instructions and reprobed with additional primary antibodies.

### 2.6. RNA Degradation Assay

SAS cells were treated under each experimental condition for 24 h, after which actinomycin D (10 μg/mL; Sigma-Aldrich, SBR00013, St. Louis, MO, USA) was added to the culture medium as previously described [8,22]. Total RNA was collected at 0, 1, 2, and 4 h after treatment, and the mRNA expression levels of *TPD52*, *TPD53*, and *TPD54* were quantified using RT-qPCR.

### 2.7. Nascent RNA Pull-Down Assay

Nascent RNA was isolated using a Click-iT Nascent RNA Capture Kit (Thermo Fisher Scientific, C10365, Waltham, MA, USA) according to the manufacturer’s instructions and a previously published report [26]. SAS cells were treated under each experimental condition for 24 h, after which 5-ethynyl uridine was added at a final concentration of 0.1 mM, and the cells were incubated for an additional 2 h to label nascent RNA. Total RNA was extracted, biotinylated by click chemistry, and captured using streptavidin magnetic beads. Newly synthesized *TPD52*, *TPD53*, and *TPD54* transcripts were quantified by RT-qPCR.

### 2.8. MTT and Caspase-3/7 Assays

At 24 h after transfection, cells were seeded into 96-well tissue culture plates at a density of 3 × 10^4^ cells/well. After overnight incubation, cells were treated under each experimental condition for 24 h. Cell viability and apoptosis were assessed using MTT assay (FUJIFILM Wako Pure Chemical Corporation, 341-01-1823, Osaka, Japan) and Caspase-Glo^®^ 3/7 assay (Promega, G8091, Madison, WI, USA) as previously described [10,23,24].

### 2.9. Invasion Assay

Cell invasion was evaluated using a Cell Invasion Assay Kit (Collagen I, ab235887; Abcam, Cambridge, UK) according to the manufacturer’s instructions. Collagen I-coated upper chambers were prepared overnight at 4 °C. Transfected cells were harvested, resuspended at 1.0 × 10^6^ cells/mL, and seeded into the upper chamber (200 μL). Medium containing a chemoattractant (kit component) was added to the lower chamber, and EGF and gefitinib were added to both chambers according to the experimental conditions. After incubation for 24 h at 37 °C, non-invading cells were removed, and invading cells were stained with cell dye. Fluorescence was measured using an Infinite M Nano+ microplate reader (TECAN, Männedorf, Switzerland; excitation, 485 nm; emission, 530 nm). Invading cells were counted using a standard curve to determine invasion rates.

### 2.10. Wound Healing Assay

The wound healing assay was performed with slight modifications to previously described methods [9,10]. Twenty-four hours after transfection, cells were seeded into 24-well tissue culture plates at a density of 4.0 × 10^5^ cells/well and cultured for 24 h. A scratch was created using a 1 mL pipette tip, and detached cells were removed by washing twice with phosphate-buffered saline. Fresh medium corresponding to each experimental condition was added, and separate wells were prepared for each time point (0, 10, and 24 h). Cells were fixed, stained with crystal violet, and imaged at each time point.

### 2.11. Cell Cycle Assay

The day after transfection, cells were reseeded into 24-well tissue culture plates at a density of 4.0 × 10^5^ cells/well. Following overnight culture, cells were treated under each experimental condition for 24 h. Cell cycle distribution was analyzed using a Tali Cell Cycle Kit (Cat. No. A10798; Thermo Fisher Scientific) and Tali Image-Based Cytometer (Thermo Fisher Scientific) according to the manufacturer’s instructions and previous reports [23,24].

### 2.12. Statistical Analysis

All experiments were performed at least three times. Data are presented as the mean ± standard deviation. Statistical analyses were performed using a one-way analysis of variance, followed by Tukey’s multiple comparison test. A two-sided *p* value < 0.05 was considered statistically significant. All statistical analyses were performed using KaleidaGraph version 4.5 (HULINKS; Tokyo, Japan). Data normality and homogeneity of variance were evaluated using the Shapiro–Wilk and Levene’s tests, respectively, prior to applying the relevant parametric statistical tests.

## 3. Results

### 3.1. EGF Regulates TPD52 Family mRNA and Protein Expression

The effects of EGF and gefitinib (an EGFR inhibitor) on the mRNA expression of TPD52 family members were examined by RT-qPCR (Figure 1). EGF significantly increased the mRNA expression of *TPD52* family members at a concentration of 1.0 ng/mL. Although EGF increased the expression of TPD52, TPD53, and TPD54, their expression levels were lower at a treatment concentration of 10 ng/mL than at 1 ng/mL, suggesting that higher concentrations of EGF do not necessarily result in greater TPD52 family mRNA expression. In contrast, gefitinib significantly increased the mRNA expression of *TPD52* and *TPD53* at a concentration of 0.5 μM, while it only had a minimal effect on *TPD54* expression. Furthermore, treatment with 0.5 μM of gefitinib abolished the EGF-induced upregulation of *TPD52* family members. To evaluate the cytotoxicity and dose response of gefitinib in SAS cells, the cell viability was measured across a range of concentrations. The half-maximal inhibitory concentration (IC_50_) of gefitinib was calculated as 6.28 µM (R^2^ = 0.897, Figure S2). Accordingly, a sub-micromolar concentration of 0.5 µM, which is well below the IC_50_ value and was expected to exert only minimal cytotoxicity, was chosen for the subsequent experiments to minimize potential cytotoxic effects, while still selectively inhibiting EGFR signaling [27].

Protein expression was subsequently evaluated by Western blotting. In contrast to the mRNA findings, EGF and gefitinib produced slight decreases in TPD52 family protein levels. These results indicate that EGF signaling regulates TPD52 family expression at both the mRNA and protein levels. Because the mRNA and protein levels differed, we next investigated the mechanisms underlying this discrepancy.

### 3.2. EGF Signaling Regulates TPD52 Family Transcriptional Activity and mRNA Stability

**Figure 2:**
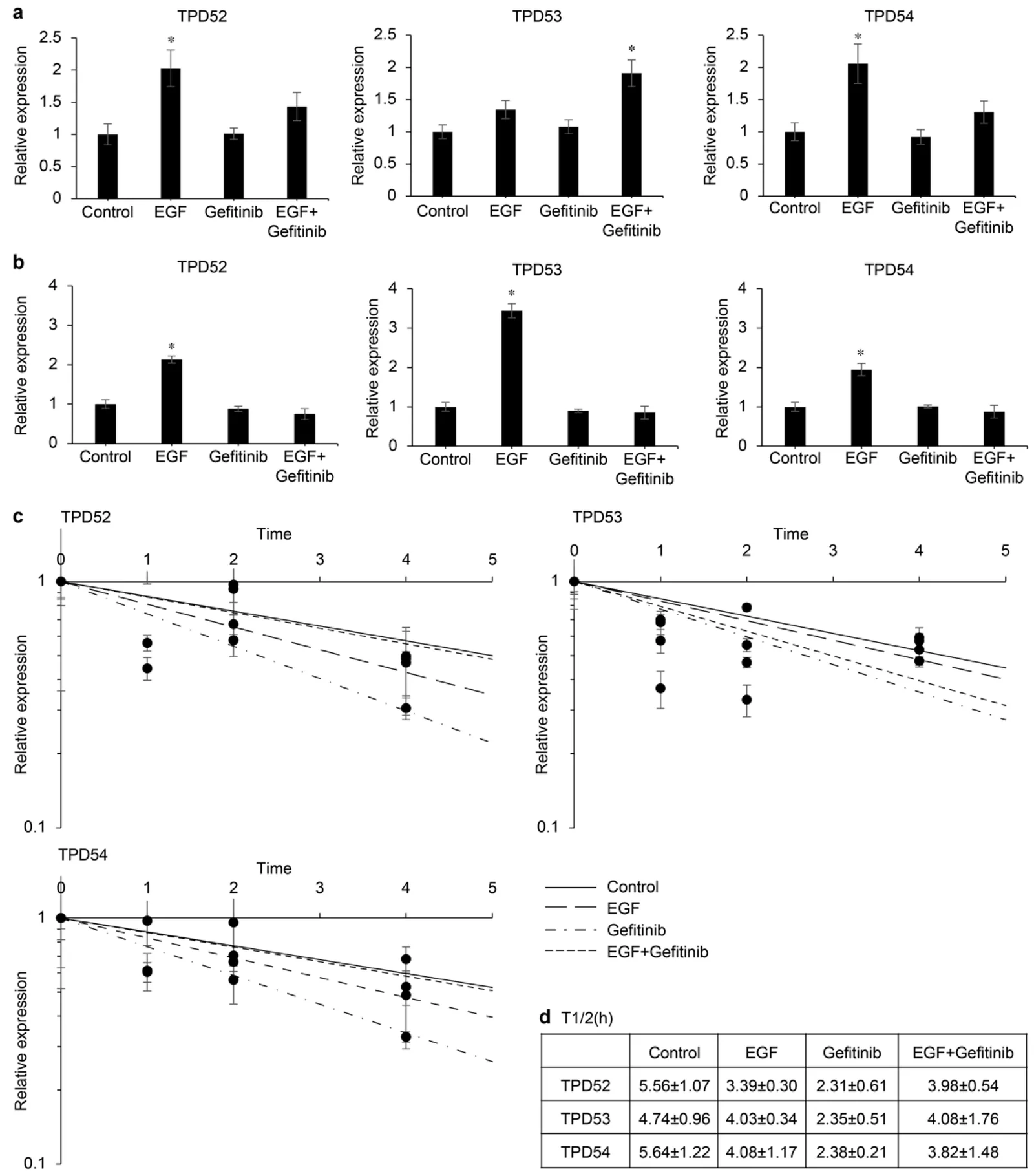
Evaluation of mRNA expression of *TPD52*, *TPD53*, and *TPD54* under different experimental conditions. (**a**) Relative nascent RNA levels by nascent RNA pull-down assay of *TPD52*, *TPD53*, and *TPD54* under different experimental conditions. (**b**) Relative steady-state mRNA levels of *TPD52*, *TPD53*, and *TPD54* under different experimental conditions. (**c**) mRNA degradation assay of *TPD52*, *TPD53*, and *TPD54*. Total RNA was collected at 0, 1, 2, and 4 h after actinomycin D treatment, and mRNA levels were measured by RT-qPCR. The value at 0 h is designated as 1, and the relative values are shown. (**d**) Estimated mRNA half-lives of *TPD52*, *TPD53*, and *TPD54* calculated by exponential curve fitting. RT-qPCR data are presented as the relative expression levels normalized to the control group (set to 1). Data are presented as the mean ± SD from three independent experiments (*n* = 3). * *p* < 0.05 versus control group. mRNA half-lives were calculated by exponential curve fitting, and representative results from three independent experiments are presented.

To investigate the mechanism underlying the increased mRNA expression of the TPD52 family, nascent RNA synthesis, steady-state mRNA expression, and mRNA stability were analyzed (Figure 2). Nascent RNA pull-down assays demonstrated that EGF markedly increased the transcription of *TPD52*, *TPD53*, and *TPD54* (Figure 2a). Consistent with these findings, RT-qPCR showed an increased steady-state mRNA expression following EGF stimulation (Figure 2b).

To evaluate mRNA stability, mRNA decay curves were generated following actinomycin D treatment, and the mRNA half-lives were calculated (Figure 2c,d). EGF shortened the mRNA half-life of all three TPD52 family members compared to the control group, and gefitinib further reduced mRNA stability. Taken together, these findings indicate that EGF simultaneously enhances transcription of TPD52 family members while accelerating mRNA turnover, suggesting that post-transcriptional mechanisms contribute to the regulation of TPD52 family expression.

### 3.3. Combined Effects of EGF and TPD52 Family Expression on Cell Viability, Apoptosis, and Autophagy

**Figure 3:**
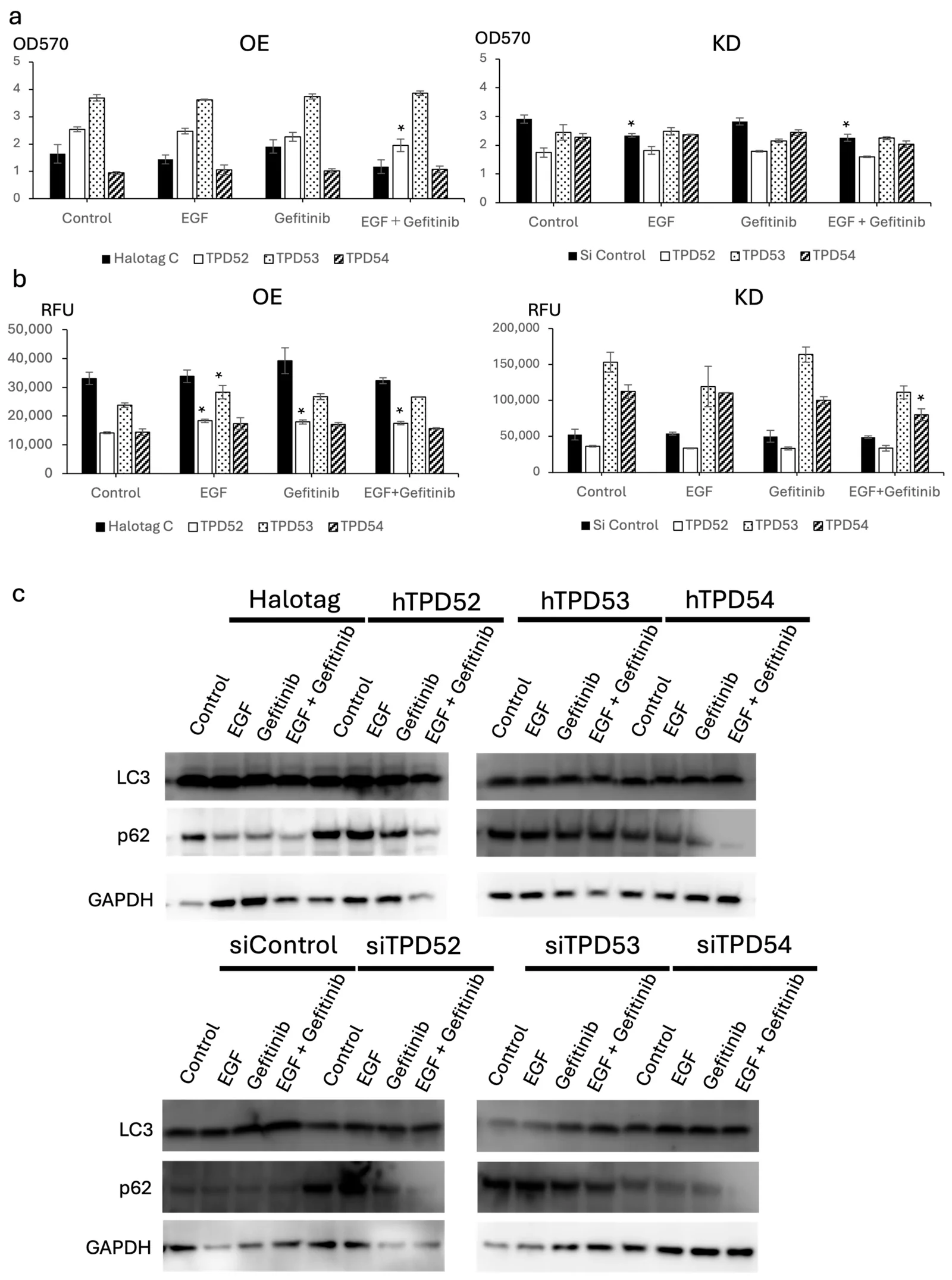
Effects of TPD52, TPD53, and TPD54 overexpression or knockdown on cell viability and cell death activity, and apoptosis under each experimental condition. (**a**) Effects on MTT assay of overexpression (OE) or knockdown (KD) of TPD52, TPD53, and TPD54. Data are presented as the absorbance at 570 nm (OD570). (**b**) Effects on caspase-3,7 activities of overexpression (OE) or knockdown (KD) of TPD52, TPD53, and TPD54. Data are presented as the relative fluorescence units (RFU). (**c**) Representative Western blot images showing the expression of LC3 and p62. Data are presented as the mean ± SD from three independent experiments (*n* = 3). * *p* < 0.05.

To investigate the combined effects of EGF signaling and TPD52 family members on cell behavior, we evaluated cell viability, apoptosis, and autophagy (Figure 3). MTT assays revealed that neither overexpression nor knockdown of *TPD52*, *TPD53*, or *TPD54* significantly (*p* > 0.05) affected cell viability under any treatment condition (Figure 3a). Similarly, caspase 3/7 activity showed little change in apoptotic activity, regardless of EGF treatment (Figure 3b). Western blot analysis of autophagy markers (LC3 and p62) revealed no consistent changes associated with TPD52 family overexpression or knockdown in either the presence or absence of EGF (Figure 3c). Densitometric analysis normalized to GAPDH further revealed that TPD54 modulation had minimal effect on LC3 and p62 levels, while TPD52 and TPD53 expression showed subtle variations under gefitinib treatment (Figure S5). These results indicate that under the present experimental conditions, modulation of TPD52 family expression had little effect on cell viability, apoptosis, or autophagy, either alone or in combination with EGF stimulation.

### 3.4. TPD54 Overexpression or Knockdown Has Little Effect on EGF- or Gefitinib-Induced Cell Invasion or Migration

**Figure 4:**
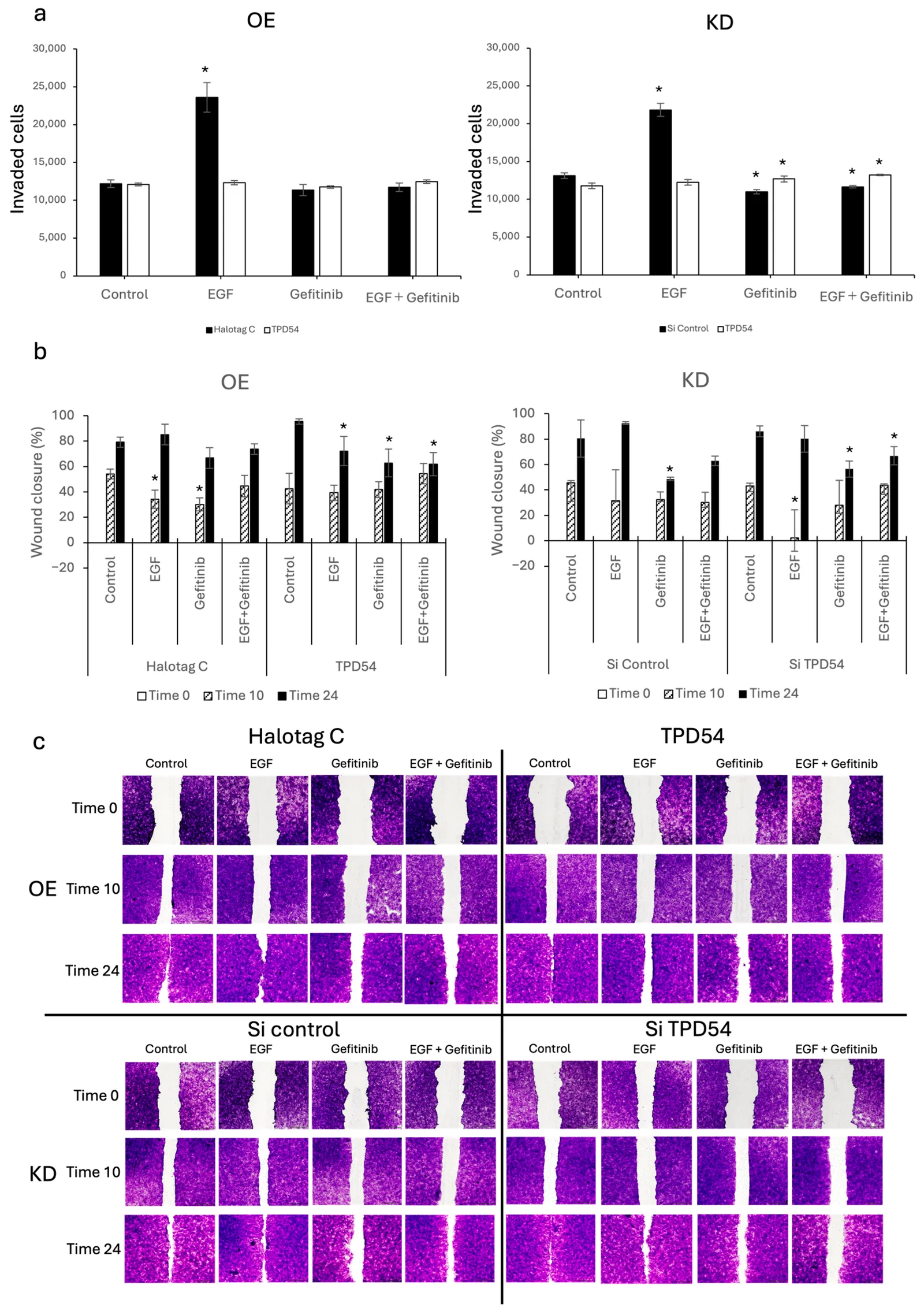
Effects of TPD54 overexpression (OE) or knockdown (KD) on cell invasion and migration. (**a**) Cell invasion assay. The invaded cells were evaluated by fluorescence intensity. Excitation wavelength, 485 nm; emission wavelength, 530 nm (for details, see Section 2). (**b**,**c**) Cell migration assay. Three microscopic images were captured for each well, and the shortest wound width in each field was measured. The percentages of wound closure at 10 and 24 h were calculated relative to 0 h using the following formula: Wound closure (%) = [(Wound width at 0 h − Wound width at 10 or 24 h)/Wound width at 0 h] × 100 (**b**). A representative image from each group is shown (**c**). Data are presented as the mean ± SD from three independent experiments (*n* = 3). * *p* < 0.05.

Next, cell invasion and migration were evaluated using invasion and wound-healing assays (Figure 4). In the invasion assay, EGF increased the invasive capacity of control SAS cells, while this increase was not clearly observed in cells with TPD54 overexpression or knockdown (Figure 4a). Similarly, the wound healing assay further showed that TPD54 overexpression or knockdown did not produce any consistent change in cell migratory activity under the different treatment conditions (Figure 4b,c). These results indicate that TPD54 had little effect on EGF- or gefitinib-induced cell invasion or migration in our experimental model.

### 3.5. TPD54 Attenuates EGF-Induced ERK Phosphorylation

**Figure 5:**
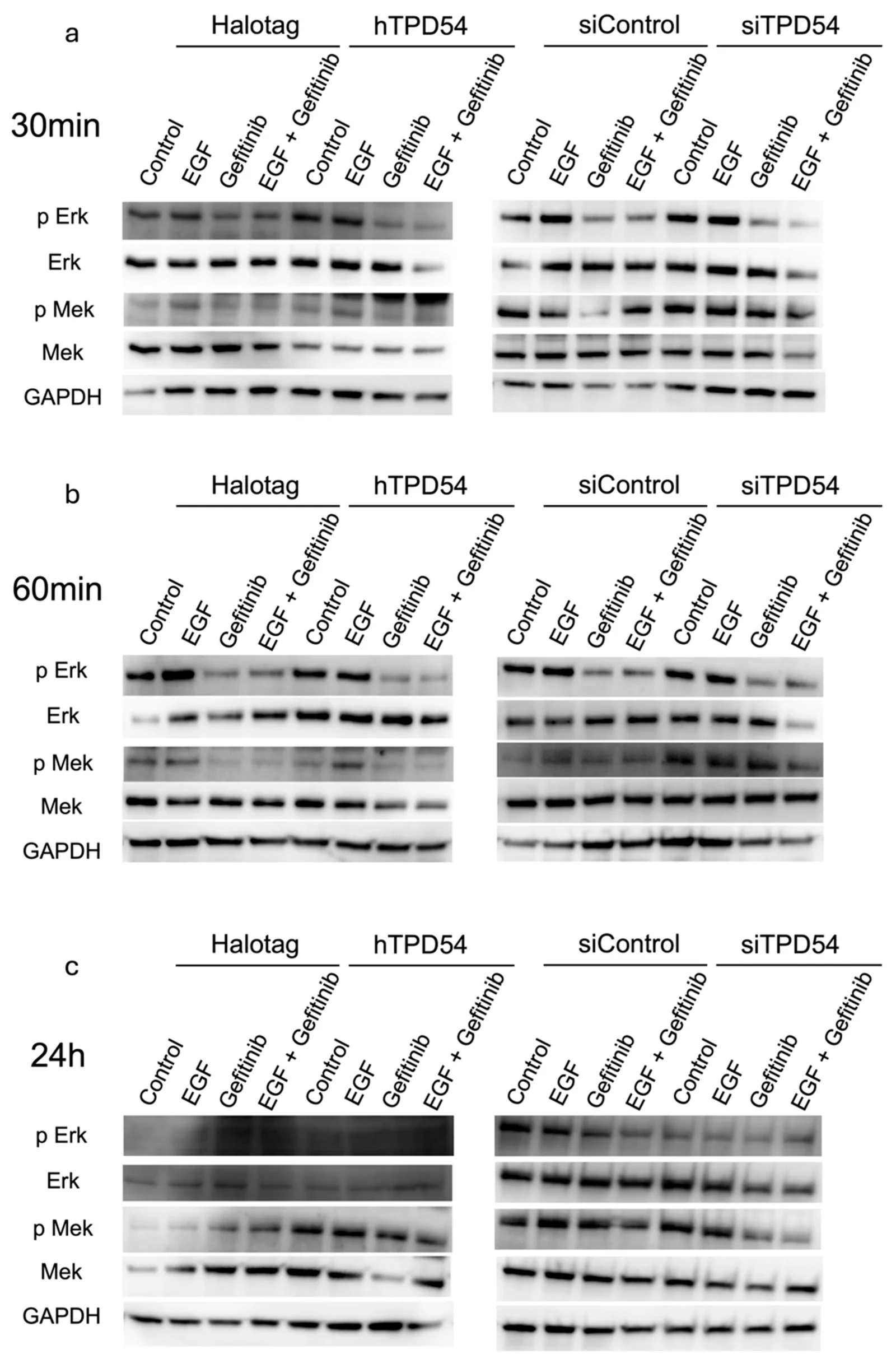
Effects of TPD54 overexpression (OE) and knockdown (KD) on MEK/ERK signaling pathways. Cells were treated under four experimental conditions (control, EGF, gefitinib, and EGF + gefitinib) as described in Materials and Methods. After 30 (**a**) and 60 min (**b**) and 24 h (**c**), cells were subjected to Western blotting for Halotag, TPD54, p-ERK, ERK1/2, p-MEK, MEK1/2 and GAPDH. Representative Western blot images of each group are shown.

The phosphorylation levels of MEK and ERK were analyzed by Western blotting (Figure 5). Thirty minutes after EGF stimulation, TPD54 overexpression reduced EGF-induced ERK phosphorylation, whereas TPD54 knockdown showed the opposite effect. These effects were most pronounced after 30 min, whereas only minor differences among groups were detected at 24 h. These findings suggest that TPD54 negatively regulates EGF-mediated ERK signaling.

### 3.6. TPD54 Regulates EGF-Induced Cell Cycle Progression

**Figure 6:**
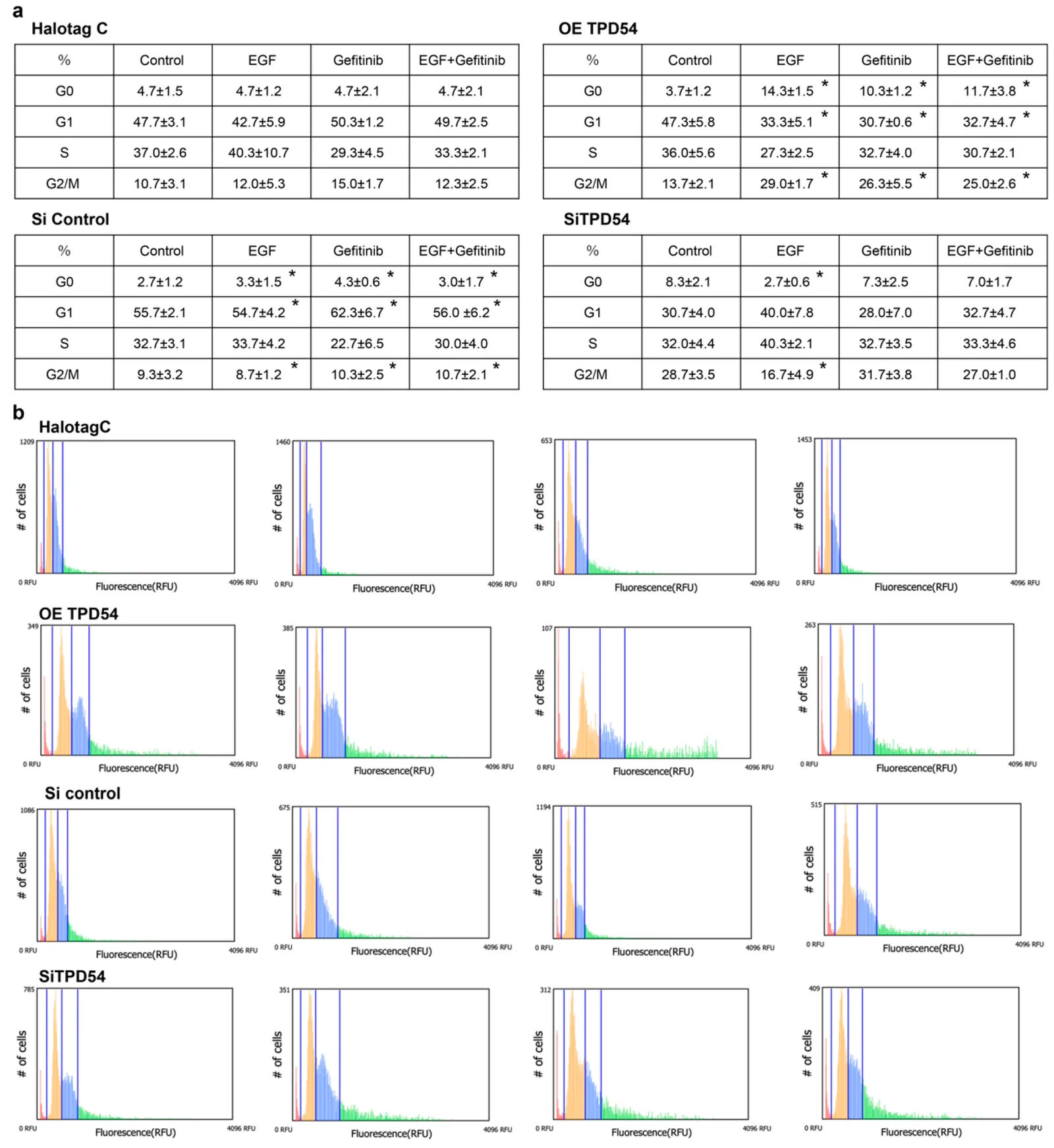
Effects of TPD54 overexpression (OE) or knockdown (KD) on cell cycle distribution. (**a**) Cell cycle distribution of cells. Data are presented as the percentages of cells in G0, G1, S, and G2/M phases. (**b**) Representative flowcytometric histogram of each group. G0, G1, S, and G2/M phases are shown in red, yellow, blue, and green, respectively. Data are presented as the mean ± SD from three independent experiments (*n* = 3). * *p* < 0.05.

The cell cycle distribution was subsequently analyzed (Figure 6). EGF treatment increased the proportion of cells in S phase. In contrast, TPD54 overexpression suppressed the EGF-induced increase in the S phase population. Furthermore, TPD54 overexpression significantly increased the proportion of cells in the G0 and G2/M phases following EGF stimulation, while TPD54 knockdown significantly decreased this proportion. These findings suggest that TPD54 negatively regulates the EGF-induced cell cycle progression, at least in part by limiting S phase entry.

## 4. Discussion

The TPD52 family is frequently overexpressed in various malignancies, including breast and prostate cancers, and has been implicated in tumor cell proliferation, survival, and metastasis [9,13,14,24,28]. Furthermore, systematic in silico and pan-cancer database analyses (e.g., of the TCGA database) have confirmed that TPD52 family members are widely upregulated across various solid tumors, where they correlate with clinical prognosis [29,30,31,32]. However, the mechanisms by which EGF signaling regulates TPD52 family expression and the role of these proteins in EGF-mediated signaling in OSCC remain unclear. Particularly, the dynamic responses of TPD52 family members at both the mRNA and protein levels following EGF stimulation and their potential involvement in feedback regulation of EGF signaling are not fully understood [24,28].

In the present study, we demonstrated that members of the TPD52 family are downstream targets of EGFR kinase activity in OSCC cells (Figure 1). We further showed that TPD54 expression was induced by EGF stimulation (Figure 1 and Figure 2) and that TPD54 subsequently attenuated EGF-induced ERK activation (Figure 5) and delayed cell cycle progression (Figure 6). These findings suggest that TPD54 functions as a negative feedback regulator that helps fine-tune EGFR signaling.

Previous studies have shown that TPD52 family members are transcriptionally regulated downstream of EGF/EGFR signaling [13,28] and that substantial post-transcriptional mechanisms, including altered mRNA stability, contribute to their expression [8,13,22]. Consistent with these observations, EGF increased the mRNA expression of all TPD52 family members (Figure 1b), while the corresponding protein levels did not increase proportionally (Figure 1d). This discordance suggests that TPD52 family expression is regulated at multiple levels, including transcription, mRNA stability, and protein turnover.

One possible explanation for this discrepancy is the presence of conserved PEST motifs within the TPD52 family proteins. PEST sequences are hydrophilic regions enriched in proline, glutamate, aspartate, serine, and threonine that promote rapid proteasomal degradation. Previous studies identified conserved PEST motifs adjacent to the N-terminal coiled-coil domain in all TPD52 family members [9,13]. Consistent with this characteristic, we observed a marked reduction in TPD52 protein expression following treatment with EGF, gefitinib, or their combination, despite induction of mRNA expression. As PEST-dependent degradation is frequently regulated by post-translational modifications, including phosphorylation [9,13], activation of EGF downstream signaling or cellular stress induced by gefitinib may enhance the recognition of these PEST motifs, thereby accelerating proteasomal degradation of TPD52 family proteins. Although this hypothesis was not directly examined, it provides one possible explanation for the discordance between mRNA and protein expression.

Interestingly, gefitinib significantly increased TPD52 and TPD53 mRNA expression, while TPD54 expression changed only minimally (Figure 1b). One possible explanation is that EGFR inhibition activates compensatory survival mechanisms through EGFR-independent pathways, such as the PI3K/Akt signaling pathway [24], thereby promoting TPD52 and TPD53 transcription [13,24]. Furthermore, the increase in TPD53 transcription observed following combined treatment with EGF and gefitinib may reflect an adaptive cellular response to maintain survival signaling despite EGFR inhibition. However, this hypothesis requires further experimental validation.

In contrast, TPD54 responded predominantly to EGF stimulation and exhibited limited induction after treatment with gefitinib alone, suggesting that TPD54 is more closely linked to EGF-dependent signaling than the other TPD52 family members. This observation provided the rationale for focusing on subsequent functional analyses on TPD54 to clarify its role in regulating EGF signaling in OSCC.

Despite attenuating ERK signaling, modulation of TPD54 expression did not significantly alter cell viability, apoptosis, proliferation, migration, or invasion under the present experimental conditions (Figure 3 and Figure 4), which may be partly attributed to the model employed. Previous studies have suggested that TPD54 regulates cell migration and adhesion through interactions with the extracellular matrix and integrin-mediated signaling [9]. As our invasion assays were performed under conventional two-dimensional culture conditions, limited extracellular matrix-dependent signaling may have masked the functional contribution of TPD54.

The inhibitory effect of TPD54 on ERK phosphorylation was most evident during the early phase after EGF stimulation and decreased over time (Figure 5). This temporal pattern suggests that compensatory signaling pathways may restore downstream signaling to promote cell survival and proliferation. Such signaling redundancy may explain why TPD54-mediated suppression of early ERK activation did not translate into marked alterations in the overall cellular phenotype.

The principal finding of the present study is that TPD54 functions as an EGF-responsive negative feedback regulator in OSCC cells. Although EGFR signaling is widely recognized as a driver of cell proliferation, our findings suggest that EGF simultaneously induces a regulatory mechanism that limits excessive signaling [33]. Specifically, TPD54 attenuated early EGF-induced ERK phosphorylation and suppressed EGF-dependent cell cycle progression, a prediction supported by the increased proportion of cells in G0 phase following TPD54 overexpression (Figure 5 and Figure 6).

Rather than completely suppressing EGFR signaling, TPD54 appears to fine-tune the magnitude and duration of EGF pathway activation. Previous studies have primarily linked TPD54 to Akt-related signaling [24], while our results indicate that it also modulates ERK signaling. Although the precise molecular mechanism remains unclear, these results identify TPD54 as a previously unrecognized component of the EGF signaling network.

Despite its suppressive effect on ERK activation and cell cycle progression, TPD54 overexpression or knockdown did not significantly alter cell proliferation, apoptosis, migration, or invasion (Figure 3 and Figure 4). One possible explanation is that the inhibitory effect of TPD54 was transient, as ERK phosphorylation largely recovered 60 min after EGF stimulation (Figure 5). Parallel survival pathways, including the PI3K/Akt pathway, may compensate for transient ERK suppression, thereby minimizing changes in the final cellular phenotype. Furthermore, previous studies suggested that TPD54-mediated regulation of cell migration and adhesion requires interactions with extracellular matrix components [9]. Therefore, the conventional two-dimensional culture system used in this study may not fully recapitulate the microenvironment required to reveal these biological effects.

**Figure 7:**
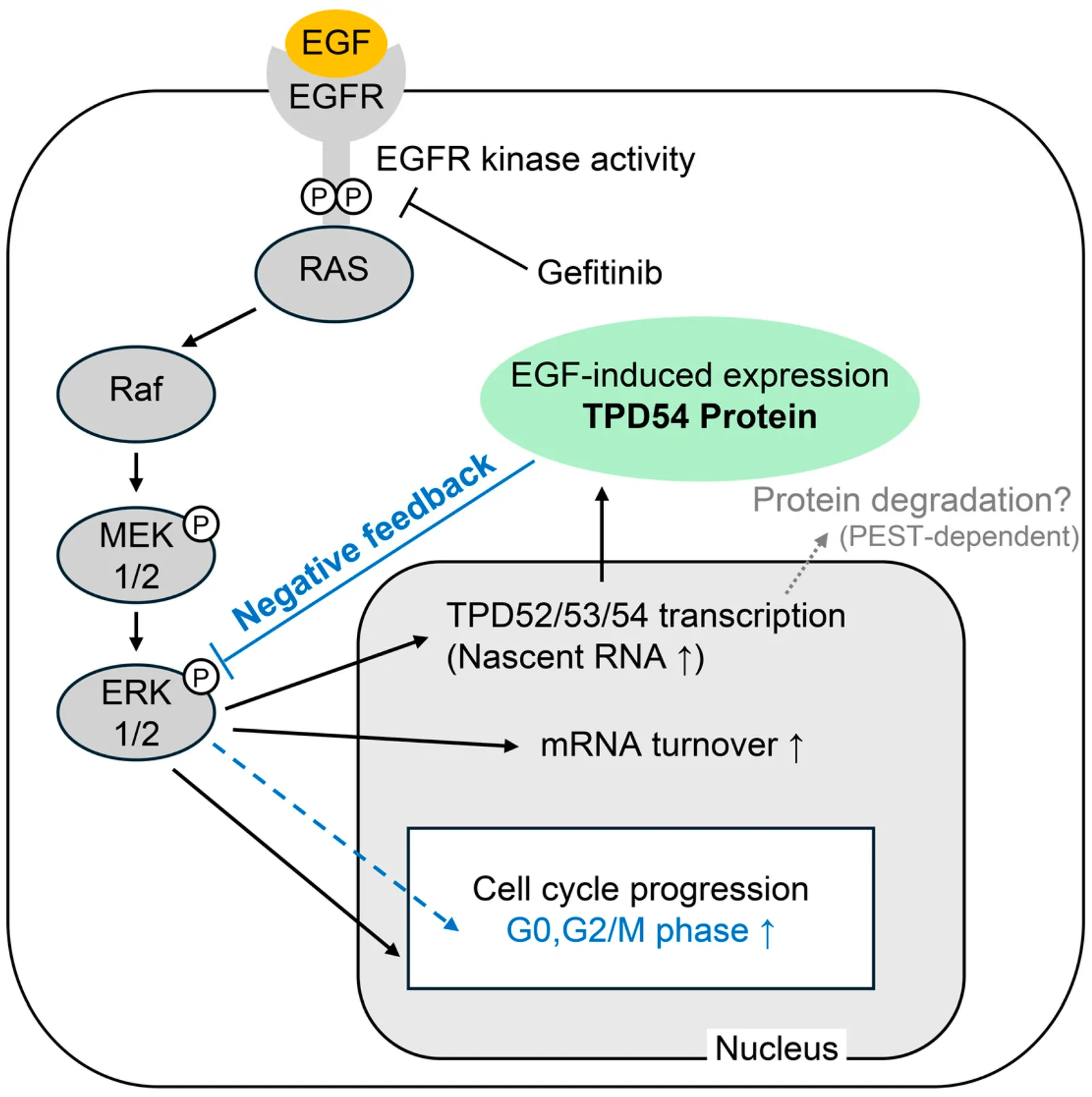
The proposed schematic model of TPD54 function in the EGF/EGFR signaling pathway in OSCC cells. EGF stimulation promotes the transcription of TPD52 family members (TPD52, TPD53, and TPD54), accompanied by accelerated mRNA turnover. The TPD54 protein functions as a negative feedback regulator by attenuating EGF-induced ERK phosphorylation, thereby promoting G0 phase accumulation in OSCC cells. Gefitinib blocks this signaling pathway by inhibiting EGFR activation.

Overall, our findings demonstrate that TPD54 is an EGF-responsive protein that attenuates ERK activation and suppresses EGF-induced cell cycle progression in OSCC cells (Figure 7). These observations suggest that TPD54 contributes to negative feedback regulation of EGF signaling, although the underlying molecular mechanisms require further investigation.

## 5. Conclusions

This study demonstrates that members of the TPD52 family are regulated downstream of EGFR signaling and identifies TPD54 as a modulator of EGFR signaling in OSCC cells. EGF-induced TPD54 expression attenuated ERK activation and suppressed EGF-induced cell cycle progression. Although TPD54 had little effect on cell proliferation, apoptosis, migration, or invasion under these specific conditions, these findings nevertheless broaden the understanding of the biological functions of the TPD52 family and identify TPD54 as a potential regulator of EGFR signaling homeostasis in OSCC.

## Figures and Tables

**Table 1:** The antibodies used in this study.

Target	Host Species	Specificity	Catalog Number	Supplier	Dilution
TPD52	Rabbit	Polyclonal	#27648-1-AP	Proteintech	1:1000
TPD53	Rabbit	Polyclonal	#14732-1-AP	Proteintech	1:1000
TPD54	Rabbit	Polyclonal	#11795-1-AP	Proteintech	1:1000
p-MEK1/2	Rabbit	Monoclonal	#2338	Cell Signaling Technology	1:1000
MEK1/2	Rabbit	Monoclonal	#9126	Cell Signaling Technology	1:1000
p-ERK1/2	Rabbit	Monoclonal	#4370	Cell Signaling Technology	1:1000
ERK1/2	Rabbit	Monoclonal	#4695	Cell Signaling Technology	1:1000
p62	Rabbit	Monoclonal	#39749	Cell Signaling Technology	1:1000
LC3	Rabbit	Monoclonal	#12741	Cell Signaling Technology	1:1000
GAPDH	Rabbit	Monoclonal	#2118	Cell Signaling Technology	1:1000
Halotag	Rabbit	Polyclonal	G9281	Promega	1:1000
Rabbit IgG	Donkey	Polyclonal	NA934V	Cytiva	1:5000

## Data Availability

The datasets generated and/or analyzed in the current study are available from the corresponding author upon reasonable request.

## Supplementary Materials

The following supporting information can be downloaded at https://www.mdpi.com/article/10.3390/biomedicines14102295/s1, Figure S1: Overview of the TPD52 family members and their roles. Schematic representation showing the homo/heterodimerization, shared cellular functions, member-specific roles in cancer progression, and specific functions of TPD52, TPD53, and TPD54 in oral squamous cell carcinoma (OSCC). Figure S2: Determination of the IC50 of gefitinib in SAS cells. SAS cells were treated with the indicated concentrations of gefitinib, and cell viability was measured using the MTT assay. Data are presented as the mean ± SD of three independent experiments. The dose–response relationship was fitted using a four-parameter logistic (4PL) model. The IC50 value was estimated from the fitted curve and was 6.28 mM. Dots represent the measured values, and the solid line represents the fitted 4PL curve. Figure S3: Validation of TPD52 family overexpression and knockdown in SAS cells. SAS cells were transfected with the HaloTag control vector, HaloTag-TPD52, HaloTag-TPD53, or HaloTag-TPD54 expression vector, or with the corresponding small interfering RNAs (siRNAs). Cells were harvested 24 h after transfection, and the expression of HaloTag, TPD52, TPD53, and TPD54 was analyzed by Western blotting. Figure S4: Validation of TPD54 overexpression and knockdown in SAS cells at different time points. SAS cells were transfected with a HaloTag control vector, HaloTag-TPD54 expression vector, or TPD54-specific small interfering RNA (siRNA). Cells were harvested at 30 min, 60 min, and 24 h after transfection, and HaloTag and TPD54 expression was analyzed by Western blotting. Figure S5: Densitometric quantification of Western blot analyses shown in Figure 1 and Figure 3. (a) Relative protein levels of TPD52, TPD53, and TPD54 in SAS cells treated with EGF, gefitinib, or both, normalized to GAPDH expression (corresponding to Figure 1d). (b) Relative protein levels of LC3 in SAS cells following TPD52, TPD53, or TPD54 overexpression (OE) or knockdown (KD) under each experimental condition, normalized to GAPDH expression (corresponding to Figure 3c). (c) Relative protein levels of p62 in SAS cells following TPD52, TPD53, or TPD54 overexpression (OE) or knockdown (KD) under each experimental condition, normalized to GAPDH expression (corresponding to Figure 3c). Band intensities were quantified using ImageJ software. All quantitative data are presented as the mean ± SD from three independent experiments (*n* = 3). Relative values were calculated by normalizing against the control group (set to 1). Figure S6: Densitometric quantification of MEK and ERK phosphorylation levels at the different time points shown in Figure 5. Densitometric quantification of MEK and ERK phosphorylation levels in SAS cells following TPD54 overexpression (OE) or knockdown (KD) under each experimental condition after (a) 30 min, (b) 60 min, and (c) 24 h of treatment (corresponding to Figure 5). Band intensities were quantified using ImageJ software. The phosphorylation levels of MEK and ERK were evaluated by calculating the ratio of phosphorylated proteins (p-MEK and p-ERK) to their respective total proteins (total MEK1/2 and total ERK1/2). All quantitative data are presented as the mean ± SD from three independent experiments (*n* = 3). Relative values were calculated by normalizing against the corresponding control group (set to 1).

## Abbreviations

The following abbreviations are used in this manuscript:

OSCC	Oral squamous cell carcinoma
EGF	Epidermal growth factor
EGFR	Epidermal growth factor receptor
MEK	Mitogen-activated protein kinase kinase
ERK	Extracellular signal-regulated kinase
GAPDH	Glyceraldehyde 3-phosphate dehydrogenase
RT-qPCR	Reverse transcription-quantitative polymerase chain reaction
SDS-PAGE	Sodium dodecyl sulfate-polyacrylamide gel electrophoresis
PVDF	Polyvinylidene difluoride
MTT	3-(4,5-dimethylthiazol-2-yl)-2,5-diphenyltetrazolium bromide
LC3	Microtubule-associated protein 1A/1B-Light chain 3
OE	Overexpression
KD	Knockdown
