# Supplementary material for: TPD54 Downregulates ERK Signaling in Oral Squamous Cell Carcinoma Cells
Source: Biomedicines. 2026 Oct 9;14(10):2295. doi: 10.3390/biomedicines14102295 (PMC13701923; doi:10.3390/biomedicines14102295)
Supplement: Supplementary file 1 [file biomedicines-14-02295-s001.zip › biomedicines-4540388-supplementary.pdf]

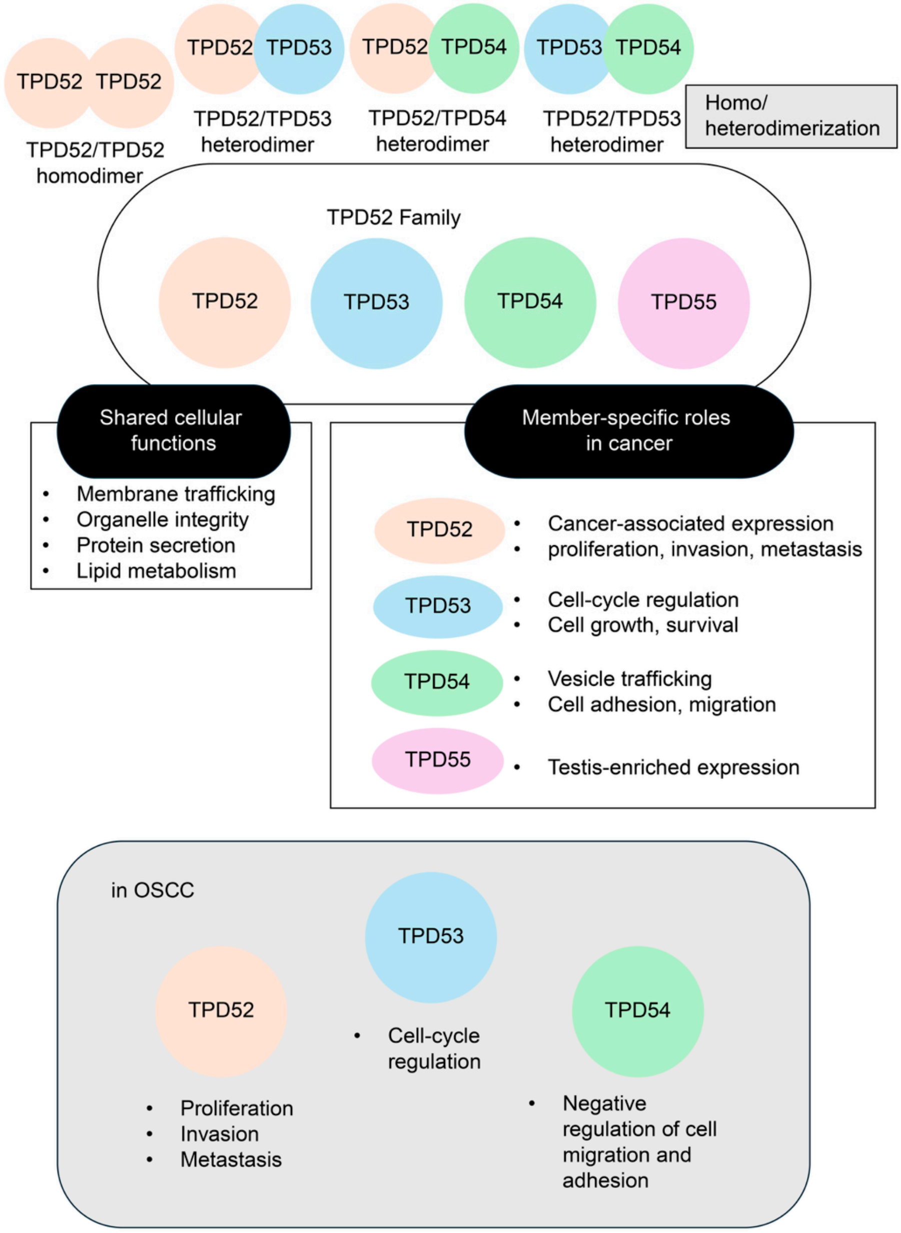

Supplementary Figure S1. Overview of the TPD52 family members and their roles. Schematic representation showing the homo/heterodimerization, shared cellular functions, member-specific roles in cancer progression, and specific functions of TPD52, TPD53, and TPD54 in oral squamous cell carcinoma (OSCC).

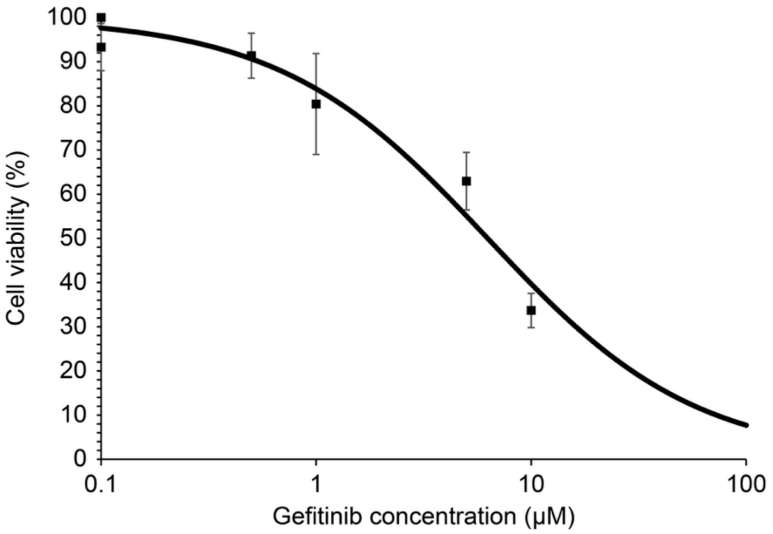

IC50	6.28 μM

Supplementary Figure S2. Determination of the IC50 of gefitinib in SAS cells. SAS cells were treated with the indicated concentrations of gefitinib, and cell viability was measured by the MTT assay. Data are presented as the mean ± SD of three independent experiments. The dose–response relationship was fitted using a four-parameter logistic (4PL) model. The IC50 value was estimated from the fitted curve and was 6.28 mM. Dots represent the measured values, and the solid line represents the fitted 4PL curve.

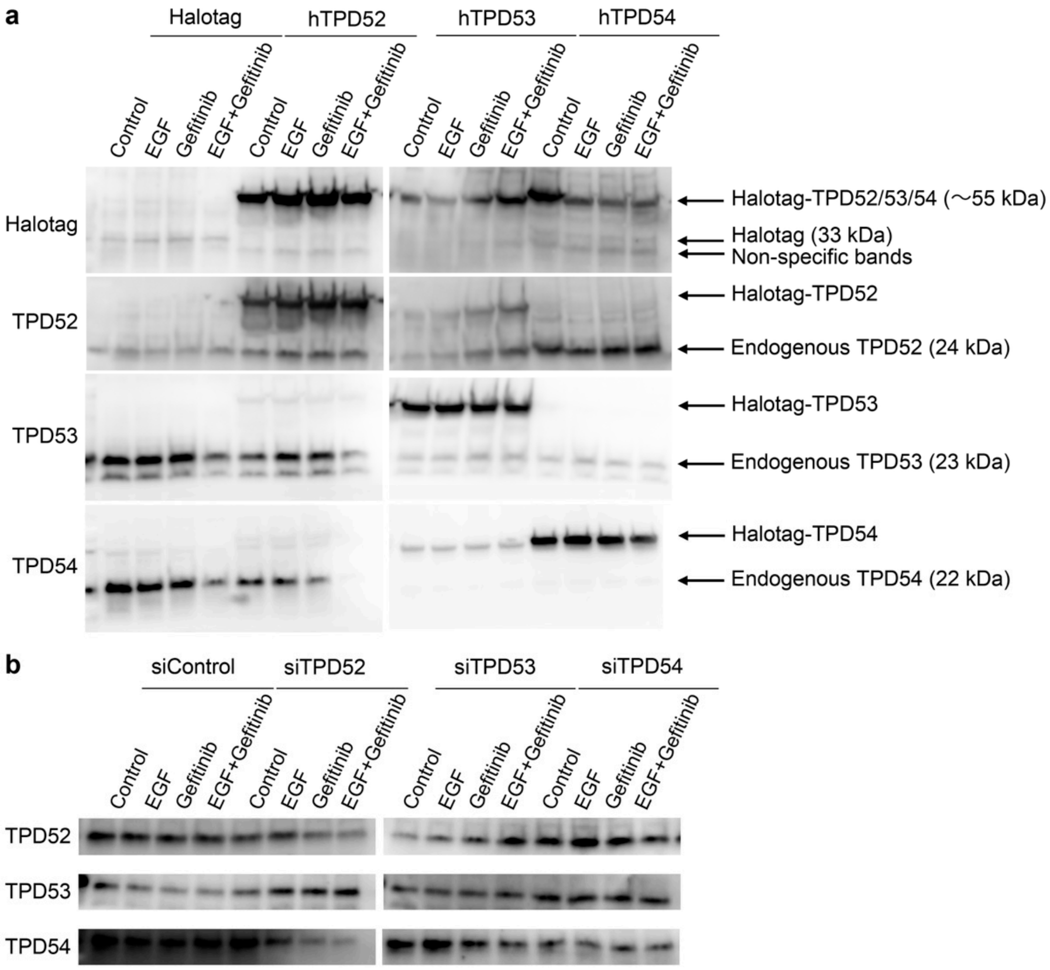

Supplementary Figure S3. Validation of TPD52 family overexpression and knockdown in SAS cells. SAS cells were transfected with the HaloTag control vector, HaloTag-TPD52, HaloTag-TPD53, or HaloTag-TPD54 expression vector, or with the corresponding small interfering RNAs (siRNAs). Cells were harvested 24 h after transfection, and the expression of HaloTag, TPD52, TPD53, and TPD54 was analyzed by Western blotting.

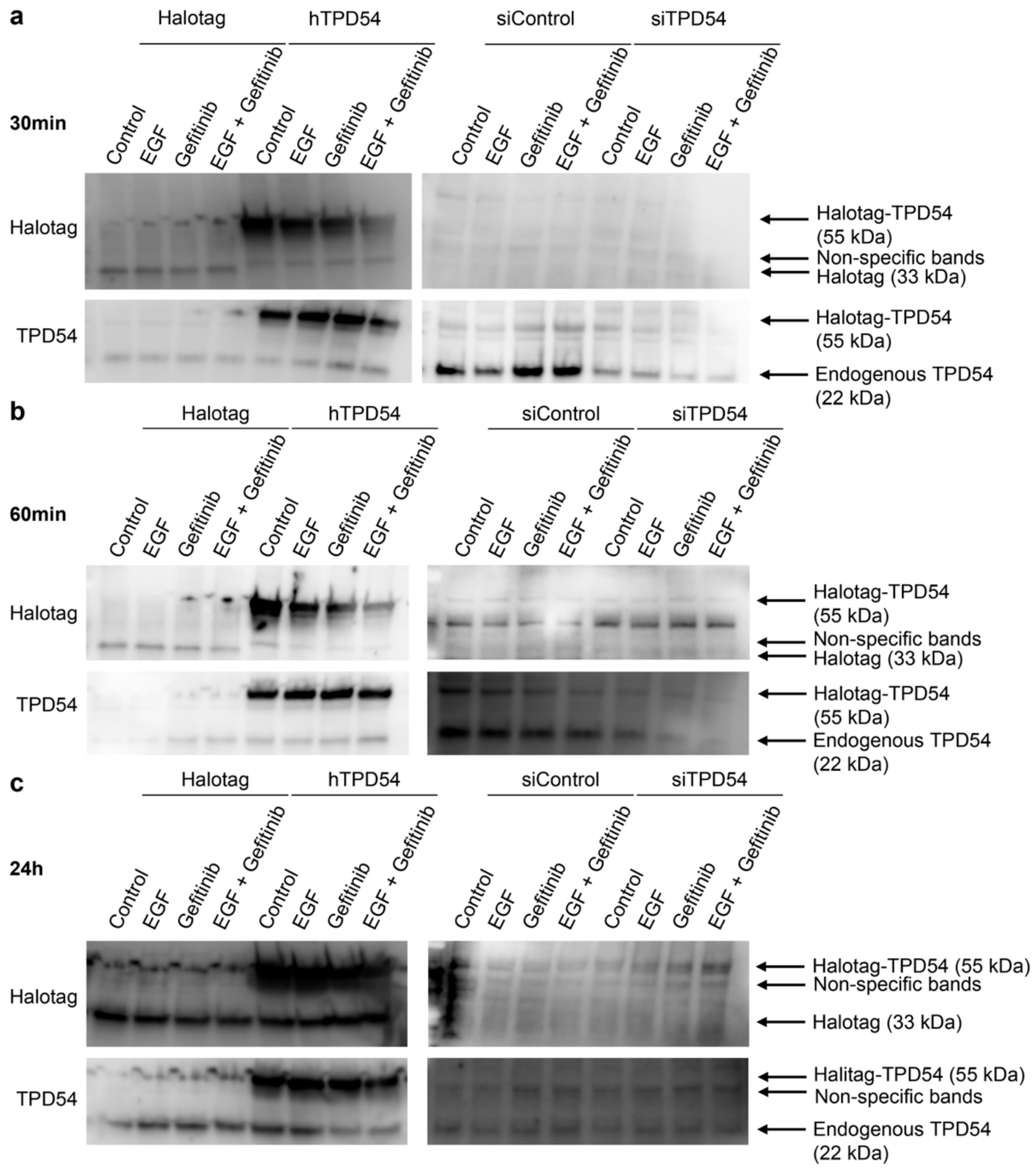

Supplementary Figure S4. Validation of TPD54 overexpression and knockdown in SAS cells at different time points. SAS cells were transfected with a HaloTag control vector, HaloTag-TPD54 expression vector, or TPD54-specific small interfering RNA (siRNA). Cells were harvested at 30 and 60 min, and 24 h after transfection, and HaloTag and TPD54 expression was analyzed by Western blotting.

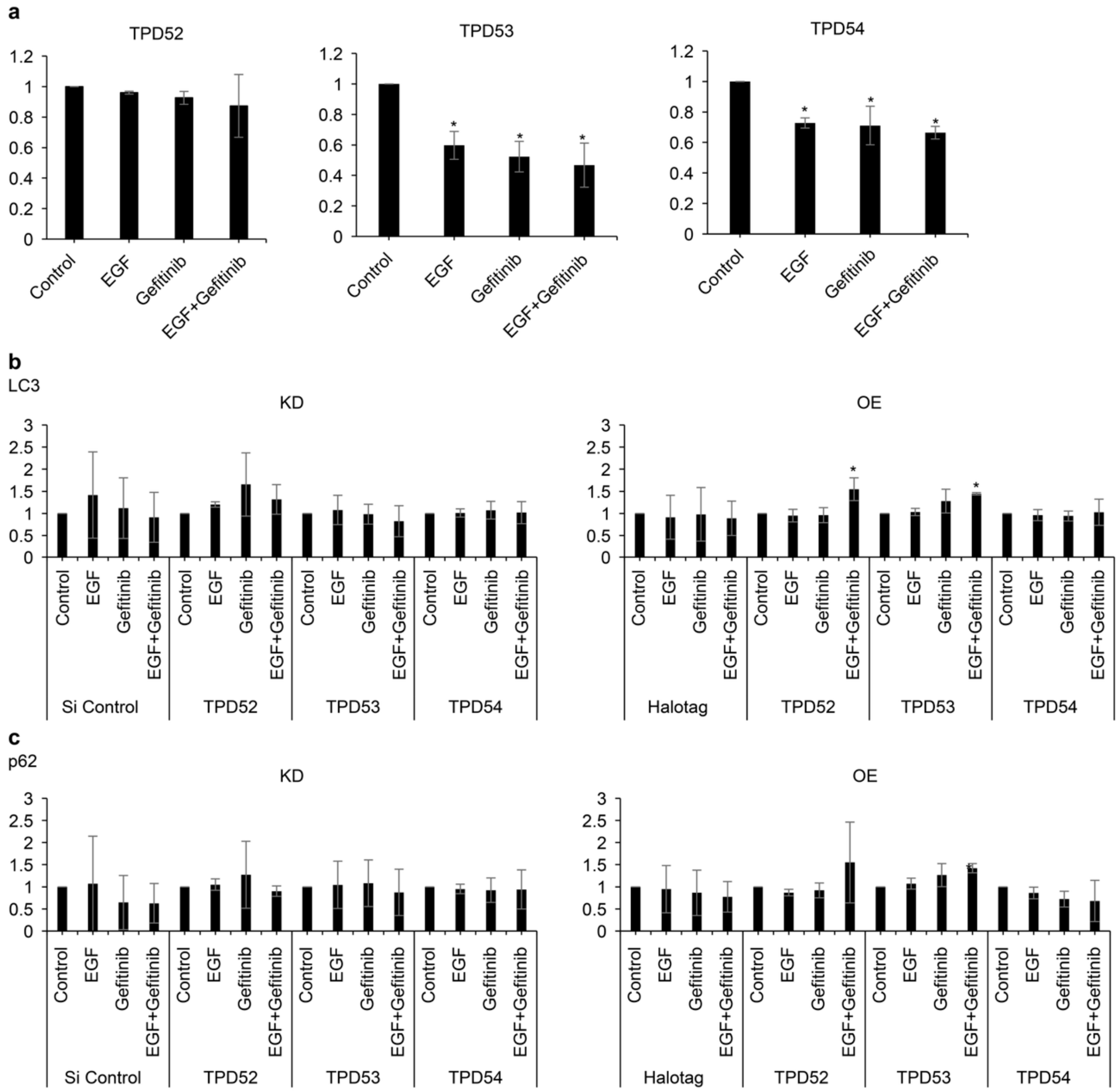

Supplementary Figure S5. Densitometric quantification of Western blot analyses shown in Figures 1 and 3. (a) Relative protein levels of TPD52, TPD53, and TPD54 in SAS cells treated with EGF, gefitinib, or both, normalized to GAPDH expression (corresponding to Figure 1d). (b) Relative protein levels of LC3 in SAS cells following TPD52, TPD53, or TPD54 overexpression (OE) or knockdown (KD) under each experimental condition, normalized to GAPDH expression (corresponding to Figure 3c). (c) Relative protein levels of p62 in SAS cells following TPD52, TPD53, or TPD54 overexpression (OE) or knockdown (KD) under each experimental condition, normalized to GAPDH expression (corresponding to Figure 3c). Band intensities were quantified using ImageJ software. All quantitative data are presented as the mean ± SD from three independent experiments (n=3). Relative values were calculated by normalizing against the control group (set to 1).

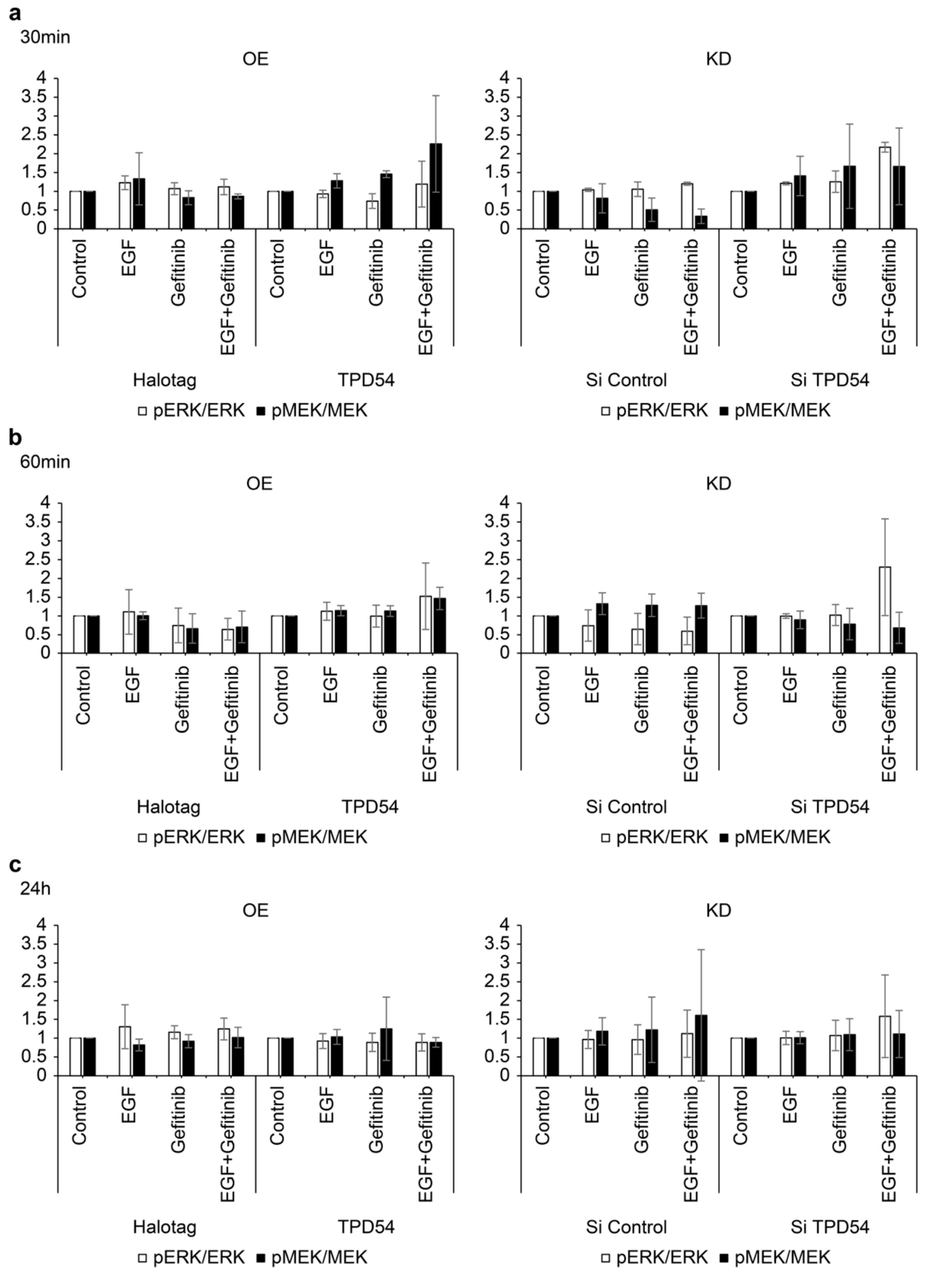

Supplementary Figure S6. Densitometric quantification of MEK and ERK phosphorylation levels at the different time points shown in Figure 5. Densitometric quantification of MEK and ERK phosphorylation levels in SAS cells following TPD54 overexpression (OE) or knockdown (KD) under each experimental condition after **(a)** 30 min, **(b)** 60 min, and **(c)** 24 h of treatment (corresponding to Figure 5). Band intensities were quantified using ImageJ software. The

phosphorylation levels of MEK and ERK were evaluated by calculating the ratio of phosphorylated proteins (p-MEK and p-ERK) to their respective total proteins (total MEK1/2 and total ERK1/2). All quantitative data are presented as the mean ± SD from three independent experiments (n=3). Relative values were calculated by normalizing against the corresponding control group (set to 1).
